# Description of two new species of Cossidae (Lepidoptera) from China

**DOI:** 10.3897/zookeys.192.2611

**Published:** 2012-05-08

**Authors:** Povilas Ivinskis, Jolanta Rimsaite, Aidas Saldaitis, Roman Yakovlev

**Affiliations:** 1 Nature Research Centre, Akademijos str. 2, LT–08412 Vilnius-21, Lithuania; 2 Altai State University, South-Siberian botanical garden, Lenina 61, Barnaul, 656049, Russia

**Keywords:** Cossidae, *Phragmataecia*, *Patoptoformis*, new species, China

## Abstract

Two new Cossidaespecies from China‘s Zhejiang and Sichuan provinces are described. The new species *Phragmataecia monika* **sp. n.**and *Patoptoformis rimsaite* **sp. n.** superficially resemble related congeners but can be distinguished by differences in wing pattern, genitalia and distribution. Checklists of the genera *Phragmataecia* and *Patoptoformis* are presented.

## Introduction

During a study of the Cossidae collection at the Zoologische Staatssammlung der Bayerischen Staaten (Munich, Germany)/Museum of Thomas Witt (Munich, Germany) the authors found two unknown specimens from China belonging to the genera *Phragmataecia* and *Patoptoformis*. After examining their morphology relative to related species the authors are describing the new species herein.

## Materials and methods

The material was collected in 2010, during May and July, using artificial light. Taxonomic nomenclature and checklists used in this study were compiled pursuant to consulting expert taxonomists and relevant literature (Schoorl 1990, Yakovlev 2011).

Abbreviations of depositories:

**ZSSM/MWM** collection of Zoologische Staatssammlung der Bayerischen Staaten (Munich, Germany)/Museum of Thomas Witt (Munich, Germany).

## Systematic accounts

### Genus Phragmataecia

Newman, 1850

http://species-id.net/wiki/Phragmataecia

Phragmataecia Newman, 1850, Zoologist 8: 2931

#### Type species.

*Noctua arundinis* Hübner, [1808]

Members of this genus are generally medium sized with very long abdomens, especially in females, and long bipectinate antennae. In males the length of pecten abruptly shortens to the distal part of tip, while in females pecten length is short to the tip of antenna as near invisible papilla. Coloration is white to black with unexpressed wing patterns except small black dots between the vein of the forewing in females.

#### Male genitalia.

Uncus base short and wide, tip acute; tegumen medium size; gnathos reduced; valvae lancete-shaped with even edges, gradually narrowing to rounded tip; juxta wide with two lateral outgrowth patches; saccus elongated, semioval form; aedeagus long, weakly hooked and slightly longer than valva; vesica without cornutus, with pale indistinct opening.

#### Female genitalia.

Long oviductus; papillae anales elongated, ellipse form; apophyses posteriores about 1.5 times longer than apophysis anterioris; ostium opening immersed, cup-like; postvaginal plate indistinct; ductus thin, long; bursa sack rounded, small without signum and with insignificant bulla on lateral side.

#### Distribution.

39 species distributed in Old world excluding Papuan and Australian ranges.

### Phragmataecia monika

Yakovlev & Saldaitis sp. n.

urn:lsid:zoobank.org:act:A8A6C34C-3A71-43D5-8F96-754BCA5B921A

http://species-id.net/wiki/Phragmataecia_monika

Figs 1–4

#### Holotype.

**Figures 1–4.**
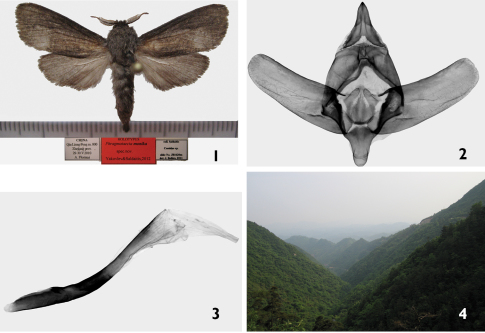
*Phragmataecia monika* Saldaitis & Yakovlev sp. n. **1** *Phragmataecia monika*, male, holotype, China, Zhejiang prov. **2** *Phragmataecia monika*, holotype, male genitalia capsule prep. Nr. UFO1 **3** *Phragmataecia monika*, holotype, male genitalia aedeagus prep. Nr. UFO1 **4** Type locality of *Phragmataecia monika*, China, Zhejiang prov.

male (Fig. 1), China, Qin Liang Feng m.800 Zhejiang prov. [province] 29-30.V.2010 A. Floriani (slide No.JB1620), (deposited in ZSSM/MWM).

#### Diagnosis.

**Figures 5–10.**
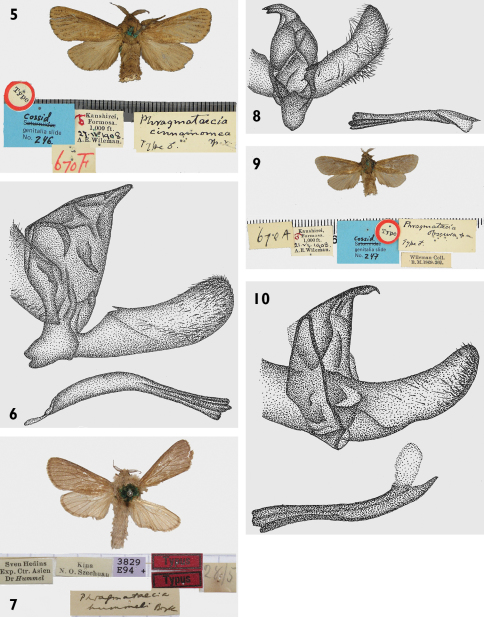
*Phragmataecia* spp., adults and genitalia. **5** *Phragmataecia cinnamomea* Wileman, adult, holotypus. **6** *Phragmataecia cinnamomea* Wileman, holotypus, male genitalia **7** *Phragmataecia hummeli* Bryk, adult, holotypus**8** *Phragmataecia hummeli* Bryk, holotypus, male genitalia **9** *Phragmataecia fusca* Wileman(*= Phragmataecia obscura* Wileman, 1911 adult **10** *Phragmataecia fusca* male genitalia.

Externallythe new speciesis most similar to sibling species *Phragmataecia cinnamomea* Wileman, 1911, *Phragmataecia hummeli* Bryk, 1942and to *Phragmataecia fusca* Wileman, 1911. *Phragmataecia cinnamomea* differs by having a yellow-brown body and wings, veins covered with dark brown scales in the postmedian forewing and dark brown dots in the terminal area (Fig. 5) and male genitalia valvae which widen to the apical part (Fig. 6). *Phragmataecia hummeli* has a grey-brownbody and unicolor wing pattern with forewings lighter brown and hindwings grey brown (Fig. 7). Its male genitalia differ by the pointed shape of the valvae, the rounded and very wide saccus and straight aedeagus (Fig. 8). *Phragmataecia fusca* has a dark yellow-brown body (Fig. 9), forewings dark brown in postmedian part, and reddish-brown hindwings. Its male genitalia valvae are rounded at the apical part (Fig. 10).

#### Description.

**Male** (Fig. 1): Forewing length of holotype 14 mm, wingspan 31 mm. Antennae one-third the length of forewing; last third strongly bipectinate with very short triangular pecten. Ground color of forewings blackish brown; median part of wing from base to inner edge yellow brown extending to j-shaped wing edge; cilia yellow mixed with brown scales; hindwing unicolor yellow, cilia greyish brown; dorsal forewing dark brown, anal edge grey; dorsal hindwing dark brown, costal area black brown. Head, thorax blackish brown.

#### Male genitalia

(Figs 2, 3): Uncus wide, strong narrowing to acute tip; valvae almost the same width as length, flat tips with long blunt outgrowths at base; tegumen wide in medial part with plunging wide neckline; saccus long, narrow, rounded; juxta wide with a pair of lateral processes; aedeagus longer than valva, thin, curved and at the tip twice wider than base.

#### Female genitalia.

Unknown.

#### Bionomics and distribution.

Known only from the Qin Liang Feng Shan mountains in Zhejiang province of eastern China (Fig. 4), *Phragmataecia monika* is likely endemic to East China. The single male specimen was attracted to light in late May at an altitude of 800m in mountainous virgin mixed forest habitat dominated by various broad-leaved trees such as oak *Quercus dentata, Quercus glauca*, poplar *Populus cathayana*, *Populus simonii*, elm *Ulmus parvifolia*, rhododendron *Rhododendron brachycarpum*, *Rhododendron dauricum*, and bamboo *Phyllostachys* spp., *Borinda* spp., *Fargesia* spp. Suspected host plantsare *Phragmites* spp.

#### Etymology.

The new species is named after Monika Rimsaite, daughter of the second author.

##### Key to species *Phragmataecia* related to *Phragmataecia monika* based on external characters

**Table:** 

1	Forewings dark colored	2
–	Forewings light colored	3
2	Forewings blackish brown	*Phragmataecia monika* sp. n., China: Zhejiang
–	Forewings dark brown	*Phragmataecia fusca* Wileman, Taiwan
3	Forewings light brown	*Phragmataecia hummeli* Bryk, China
–	Forewings yellow brown with dark brown scales group and dots in terminal part	*Phragmataecia cinnamomea* Wileman, China, Taiwan

##### Key to species *Phragmataecia* related to *Phragmataecia monika* based on male genitalia

**Table:** 

1	Saccus long rounded	2
–	Saccus short or slightly bilobed	3
2	Valva almost the same width as length	*Phragmataecia monika* sp. n., China: Zhejiang
–	Valva in the apical part pointed	*Phragmataecia hummeli* Bryk, China
3	Valva rounded, wider in the apical part	*Phragmataecia cinnamomea* Wileman, China, Taiwan
–	Valva in the apical part slightly narrowed, rounded	*Phragmataecia fusca* Wileman, Taiwan

### Genus Patoptoformis

Yakovlev, 2006

http://species-id.net/wiki/Patoptoformis

Patoptoformis Yakovlev, 2006, Tinea, 19 (3): 203.

#### Type species.

*Patoptoformis hanuman* Yakovlev, 2006.

Small dark colored moths with dark hair densely covering the body. Antennae bipectinate. Forewing with a scarcely seen streaky pattern; hindwing dark without pattern; fringe evenly dark on both wings. Sexual dimorphism weakly expressed but female somewhat larger than male with wider wings and non-pectinate antennae.

#### Male genitalia.

Uncus long, narrowly triangular with pointed apex; gnathos arms long and densely covered with spinules; valva with costal crest, blunt apex and scarcely noticeable transition between sclerotized and membranous parts, sclerotization gradually weakening towards apex; arms of transtilla small, pointed; juxta small; saccus very poorly expressed; aedeagus short, vesica opening occupies a dorso-apical position and comprises half of aedeagus length; vesica without cornuti.

#### Female genitalia.

Papillae anales elongated with rounded apices; apophyses posteriores thin, twice as long as anteriores; ostium opening immersed, fissure-like, surrounded by cordate rim; ductus bursae membranous, long and narrow; bursa elongate, gradually inflating to apex; ductus seminalis thin, enters bursa near its junction with ductus bursae.

#### Distribution.

Three species distributed in NE India (Assam), Nepal, SE China (Sichuan).

### Patoptoformis rimsaitae

Saldaitis & Yakovlev sp. n.

urn:lsid:zoobank.org:act:2F9D4618-3ED3-454E-A454-65B85A0D89EB

http://species-id.net/wiki/Patoptoformis_rimsaitae

Figs 11–14

#### Type material.

**Holotype**: male China, Sichuan prov. [province], Env. [environs] Mianning Ling Shan Mts. [mountains], h[high], -3760 m 01-03. 07. 2010, local collector leg. (slide No.JB1620), (deposited in ZSSM/MWM).

#### Diagnosis.

Externallythe new speciesis most similar to sibling species *Patoptoformis ganesha* (Yakovlev, 2004) and *Patoptoformis hanuman* Yakovlev, 2006. Unlike the new species, *Patoptoformis ganesha* has dark forewings generally with a row of narrow transversal bands in medial and submarginal zones and black hindwings with a black fringe (Fig. 15). Male genitalia in *Patoptoformis ganesha* differ as its uncus is triangular, broad gnathos is densely set with spinules, valvae are cut near apex, arms of transtilla are hook-like and saccus is rounded (Fig. 16). In *Patoptoformis hanuman* the forewings are brown with a faint black streaky pattern with a clear submarginal streak and spot in the distal area and hindwings are dark brown, almost black (Figs 17, 19). Male genitalia differ in shape of the valvae which are narrower, the gnathos arms which are thicker and aedeagus which is somewhat curved in the middle (Fig. 18).

**Figures 11–14.**
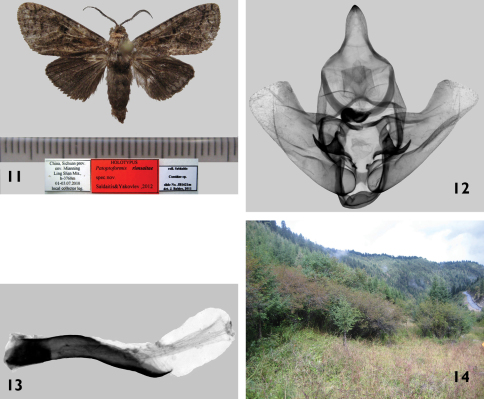
*Patoptoformis rimsaitae* Saldaitis & Yakovlev sp. n. **11** *Patoptoformis rimsaitae* Saldaitis & Yakovlev, holotype, Sichuan province **12** *Patoptoformis rimsaitae*, holotype, male genitalia, capsule, prep. Nr. UFO2 **13** *Patoptoformis rimsaitae*, holotype, male genitalia, aedeagus, prep. Nr. UFO2 **14** Type locality *Patoptoformis rimsaitae*, China, Sichuan.

**Figures 15–19.**
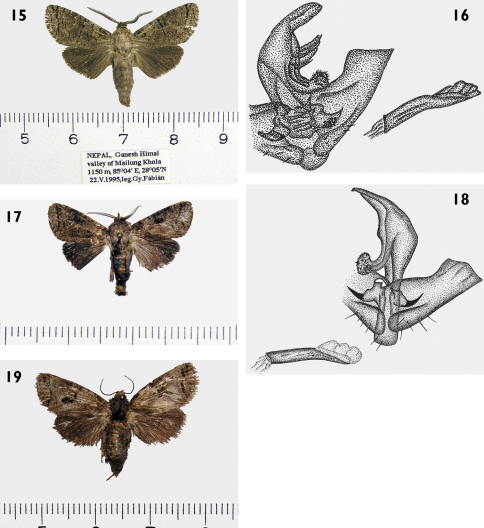
*Patoptoformis* spp. adults and genitalia. **15** *Patoptoformis ganesha* (Yakovlev,2004, holotypus **16** *Patoptoformis ganesha*, holotypus male genitalia **17** *Patoptoformis hanuman* Yakovlev,2006 holotypus, male **18** *Patoptoformis hanuman*, holotypus, male genitalia **19** *Patoptoformis hanuman*, paratypus, female.

#### Description.

**Male** (Fig. 11): Forewing length of holotype 11 mm; wingspan 24 mm. Antennae almost half as long as forewing, strongly bipectinate and last third with very short triangular pecten; ground color of forewings grey blackish with large yellow patch in middle of basal area, middle part of wing from base to inner edge yellow brown, cilia yellow mixed with brown scales; hindwing unicolor yellow, cilia greyish brown; upper side of forewing dark brown, anal edge grey; upper side of hindwing dark brown, costal area black brown; head, thorax blackish brown.

#### Male genitalia

(Figs 12, 13): Uncus wide with blunt tip; gnathos wide but arms narrow; valvae short, very wide at base narrowing to middle then widening abruptly at tip; arms of transtilla hook-like, thin, acute in tip; saccus wide, rounded; aedeagus almost the same length as valva, weakly curved with sharp curved tip; vesica like equilateral sack with opening size more than half of aedeagus length.

#### Female genitalia.

Unknown**.**

#### Bionomics and distribution.

Known only from the China’s Sichuan province on the eastern edge of the Tibetan plateau. *Patoptoformis rimsaitae*is likely endemic to West Sichuan. A single male was attracted to light at an altitude of 3700 m. The new species was collected in the shrubby transition between the mountain primary mixed forest and the alpine grassland zones (Fig. 14). Nothing is known about the early stages.

#### Etymology.

The species is named in honor of Dr Jolanta Rimsaite, a prominent expert of general entomology.

##### Key to species *Patoptoformis* based on external characters

**Table:** 

1	Forewings dark brow with reticulated patterns formed by black lines	2
–	Forewings dark brown without reticulated patterns but with big black patch	*Patoptoformis hanuman* Yakovlev, India: Assam
2	Forewings grey black with yellow patch	*Patoptoformis rimsaitae* sp. n., China: Sichuan
–	Forewings with row of narrow transversal bands in medial and submarginal zones	*Patoptoformis ganesha* (Yakovlev), Nepal

##### Key to species *Patoptoformis* based on male genitalia

**Table:** 

1	Arms of transtilla hook –like, thin	2
–	Arms of transtilla hook –like, massive	*Patoptoformis hanuman* Yakovlev, India: Assam
2	Tip of valva flat, edges rounded	*Patoptoformis rimsaitae* sp. n., China: Sichuan
–	Tip of valva with pointed edge	*Patoptoformis ganesha* (Yakovlev), Nepal

##### Checklist of the genus *Phragmataecia*

***Phragmataecia albida* Erschoff, 1874**

=*Pragmataecia erschoffi* Reisser, 1962

Distribution. Iran, Turkmenistan, Uzbekistan, Kazakhstan, NW China (Kuldja), Afghanistan, SW Russia (S. Volga reg.) (Christoph 1884, Daniel 1963, 1965, Falkovitch 1986, Gross 1925, Uvarov 1910, Yakovlev 2005a, 2009a).

***Phragmataecia andarana* Clench, 1959**

Distribution: Namibia, South Africa (Vári et al. 2002).

***Phragmataecia anikini* Yakovlev, 2011**

Distribution: SW Mongolia (Hovd aimak, Dzhungarian Gobi desert) (Yakovlev 2011).

***Phragmataecia annapurna* Yakovlev, 2009**

Distribution: Nepal (Annapurna Himal) (Yakovlev 2009a).

***Phragmataecia brunni* Pagenstecher, 1892**

Distribution: E. Africa (Tanzania) (Pagenstecher 1892).

***Phragmataecia castaneae* (Hübner, 1790)**

= *Phalena (Bombyx) arundinis* Hübner [1802-1808]

= *Phalena castanea*, Esper (1807)

= *Phragmatoecia castanea* Teich, 1884

= *Phragmataecia castanea sicca* Dannehl, 1829

= *Phragmataecia castaneae f. fusca* Lempke, 1961

= *Phragmataecia castaneae leonadae* Gomez Bustillo, 1977

= *Phragmataecia meloina* Gomez Bustillo & Fernandes-Rubio, 1976

= *Phragmataecia sica* Gomez bustillo & Fernandes-Rubio, 1976

Distribution: Central and Southern Europe, S. England, M. East, Caucasus, Transcaucasia, Turkmenistan, Kazakhstan, NW Iran, Iraq, Syria, Lebanon, Turkey, W. China, SW Siberia, Egypt, Tunisia, Morocco (Yakovlev 2011).

***Phragmataecia cinnamomea* Wileman, 1911**

= *Xyleutes Hansi* Strand, 1915.

Distribution: Taiwan, S. China (Jianxi-Fujian border) (Gaede 1933, Ueda in Heppner and Inoue 1992, Wang and Lee 1998, Yakovlev 2009b).

***Phragmataecia dushman* Yakovlev, 2009**

Distribution: Afghanistan (Yakovlev 2009a).

***Phragmataecia furia* Grum-Grshimailo, 1890**

Distribution: Uzbekistan, Tadzhikistan ?, Afghanistan (Daniel 1964).

***Phragmataecia geisha* Yakovlev, 2011**

Distribution: Japan (Yakovlev 2011).

***Phragmataecia gummata* Swinhoe, 1892**

= *Phragmatoecia* (sic!) *lata* Snellen, 1895

= *Phragmatoecia* (sic!) *sordida* Snellen, 1901

Distribution: China (Fukien, Lingping), Vietnam, Thailand, Indonesia (Java, Sumatra) (Daniel 1940, Gaede 1933, 1949, Roepke 1957, Yakovlev 2009b, Yakovlev and Witt 2009).

***Phragmataecia gurkoi* Yakovlev, 2007**

Distribution: NW Pakistan (Yakovlev 2007a).

***Phragmataecia fusca* Wileman, 1911**

= *Phragmataecia obscura* Wileman, 1911

Distribution: Taiwan (Ueda 1992), Thailand, Hong Kong (Ades and Kendrick 2004).

***Phragmataecia fuscifusa* Hampson, 1910**

Distribution: Sierra Leone, Nigeria (Yakovlev 2011).

***Phragmataecia hummeli* Bryk, 1942**

Distribution: China (NE Sichuan) (Yakovlev 2009b).

***Phragmataecia impura* Hampson, 1891**

Distribution: India, Nepal, S. China (Hainan Isl., Zhejiang and Guangxi prov.), Vietnam, Laos, Thailand, Java (Snellen 1901, de Joannis 1929, Arora 1976, Yakovlev 2004, 2009b, Yakovlev and Witt 2009).

***Phragmataecia innominata* Dalla Torre, 1923**

= *Phragmatoecia reticulata* Hampson, 1910

Distribution: South Africa, Mozambique, Malawi (Schoorl 1990, Vári et al. 2002).

***Phragmataecia innotata* (Walker, 1865)**

Distribution: China, Vietnam, Laos, Thailand (Yakovlev and Witt 2009, Yakovlev 2011).

***Phragmataecia irrorata* Hampson, 1910**

Distribution: Zimbabwe, South Africa, Namibia, Bostwana, Mozambique, Zambia, Malawi (Pinhey 1979, Vári et al. 2002, Yakovlev 2011).

***Phragmataecia itremo* Viette, 1974**

Distribution: Madagascar (Viette 1974).

***Phragmataecia laszloi* Yakovlev, 2009**

Distribution: Nepal (Annapurna Himal) (Yakovlev 2009a).

***Phragmataecia longivitta* Candeze, 1926**

Distribution: Laos (Ćandèze 1926).

***Phragmataecia minima* Hampson, 1891**

Distribution: S. India (Hampson 1891).

***Phragmataecia minor* Moore, 1879**

Distribution: Bangladesh, Myanmar ?, China (Lingping) (Cotes, Swinhoe 1887, Swinhoe 1890, Daniel 1949, Yakovlev 2011).

***Phragmataecia monika* Saldaitis & Yakovlev, sp. n.**

Distribution: China Zhejiang province.

***Phragmataecia okovangae* Clench, 1959**

Distribution: Namibia, South Africa (Vári et al. 2002).

***Phragmataecia pacifica* Yakovlev, 2007**

Distribution: Russia, Caucasus, Daghestan (Yakovlev 2007b).

***Phragmataecia parvipuncta* (Hampson, 1892)**

Distribution: India, Sri Lanka, Vietnam (Arora 1976, Gaede 1933, de Joannis 1929, Yakovlev and Witt 2009).

***Phragmataecia pelostema* (Hering, 1923)**

Distribution: Togo, Cameroon, Nigeria (Yakovlev 2011).

***Phragmataecia pectinicornis* (Strand, 1914)**

Distribution: Central Sudan (Strand 1914).

***Phragmataecia psyche* (Le Cerf, 1919)**

Distribution: Benin? and different parts of Western Africa (Yakovlev 2011).

***Phragmataecia purpureu*s Fletcher, 1927**

Distribution: India (Bihar) (Arora 1976; Fletcher 1927).

***Phragmataecia pygmaea* Graeser, 1888**

Distribution: SE Russia, Korea, NE China (Charbin) (Staudinger 1892, Staudinger & Rebel 1901, Witt 1985, Yakovlev 2005b, 2009b).

***Phragmataecia roborowskii* Alpheraky, 1897**

= *Phragmataecia longialatus* Hua, Chou, Fang & Chen, 1990

Distribution: NW China, S. Mongolia (Yakovlev 2007c).

***Phragmataecia saccharum* Moore, 1879 (Walker, 1865)**

Distribution: India (Cotes and Swinhoe 1887).

***Phragmataecia sericeata* Hampson, 1910**

Distribution: Ghana, Nigeria (Yakovlev 2011).

***Phragmataecia sumatrensis* Snellen, 1892**

Distribution: Indonesia (Sumatra) (Snellen 1892, Gaede 1933).

***Phragmataecia terebrifer* Fletcher, 1927**

Distribution: India (Fletcher 1927).

***Phragmataecia turkmenbashi* Yakovlev, 2008**

Distribution: Turkmenistan (Kopetdagh Mts., Valley of Ipay-Kala ) (Yakovlev 2008).

##### Checklist of the genus *Patoptoformis*

***Patoptoformis ganesha* (Yakovlev, 2004)**

Distribution: Nepal, Ganesh Himal.

***Patoptoformis hanuman* Yakovlev, 2006**

Distribution: NE India, Assam.

***Patoptoformis rimsaitae* Saldaitis &Yakovlev, sp. n.**

Distribution: province China, Sichuan.

## Supplementary Material

XML Treatment for Phragmataecia

XML Treatment for Phragmataecia monika

XML Treatment for Patoptoformis

XML Treatment for Patoptoformis rimsaitae
